# Body Composition, Fasting Blood Glucose and Lipidemic Indices Are Not Primarily Determined by the Nutritional Intake of Middle-Aged Endurance Trained Men—Another “Athletes’ Paradox”?

**DOI:** 10.3390/jcm11206057

**Published:** 2022-10-13

**Authors:** Spyridon Methenitis, Konstantinos Feidantsis, Athina Kaprara, Apostolos Hatzitolios, Petros Skepastianos, Sousana K. Papadopoulou, George Panayiotou

**Affiliations:** 1 Sports Performance Laboratory, School of Physical Education & Sports Science, National and Kapodistrian University of Athens, GR-17237 Athens, Greece; 2 Department of Nutrition Sciences and Dietetics, Faculty of Health Sciences, International Hellenic University, GR-57400 Thessaloniki, Greece; 3 Theseus, Physical Medicine and Rehabilitation Center, GR-17161 Athens, Greece; 4 Laboratory of Animal Physiology, Department of Zoology, School of Biology, Aristotle University of Thessaloniki, GR-54124 Thessaloniki, Greece; 5 Laboratory of Sports Med, School of Physical Education and Sports Science, Aristotle University of Thessaloniki, GR-54124 Thessaloniki, Greece; 6 1st Department of Cardiology, AHEPA Hospital, Aristotle University of Thessaloniki, GR-54124 Thessaloniki, Greece; 7 Department of Biomedical Sciences, Faculty of Health Sciences, International Hellenic University, GR-57400 Thessaloniki, Greece; 8 Department of Life Sciences, School of Sciences, European University Cyprus, Nicosia CY-1516, Cyprus

**Keywords:** runners, body fat, lean body mass, oxidative metabolism, cardiovascular risk factors, energy expenditure, energy balance

## Abstract

Systematic, regular high-volume endurance training induces significant metabolic adaptations in glucose and lipids metabolism, which seems to affect the negative impact of unhealthy nutrition, at least in animal models. The present study aimed to investigate the main determinants of body composition, blood glucose and lipids concentrations between middle-aged sedentary individuals (Sed) and well-trained endurance athletes (Run), both following an unhealthy high-fat diet. In thirty-five Sed (Age: 54.0 ± 6.6 yrs, Body Mass: 77.1 ± 10.5 kg, BMI: 31.3 ± 6.0 kg·m^−2^) and thirty-six Run (Age: 51.6 ± 5.2 yrs, Body Mass: 85.8 ± 3.4 kg, BMI: 23.2 ± 1.8 kg·m^−2^), body composition, nutritional intake, energy expenditure, resting metabolic rate (RMR), respiratory exchange ratio (RER), and blood glucose and lipids concentrations were evaluated. Multiple linear regression analyses revealed that body composition, blood glucose and lipids’ concentrations in the Run group were primarily determined by the energy expenditure (B: −0.879 to −1.254), while in the Sed group, by their energy intake (B:−0.754 to 0.724). In conclusion, it seems that in well-trained endurance middle-aged athletes, body composition, blood glucose, and lipids concentrations seem to be determined by their training-induced daily energy expenditure and not by their nutritional intake per se. At the same time, nutrition is the primary determinant in aged-matched sedentary individuals, even if they both follow high-fat diets.

## 1. Introduction

The prevalence of overweight, obesity and dyslipidemia is reaching epidemic proportions worldwide [1]. As a result, a growing number of predominantly middle-aged (from 40 to 60 years old) people suffer from increased metabolic disorders/dysfunctions and cardiometabolic risk factors/chronic diseases [2,3]. Although caloric-rich nutrition and high-fat diets are considered to be two of the major determinants of increased body fat mass, obesity, dyslipidemia, insulin resistance and diabetes in sedentary individuals [2,4,5,6], it seems that nutrition alone cannot explain the significant inter-individual variations of the response to unhealthy diet/nutrition. e.g., nutrition with more than 30% and 10% of daily energy intake from fats and saturated fatty acids, respectively [7], and thus of diet-induced obesity, dyslipidemia, insulin resistance and diabetes [5,6]. Specifically, genetic and epigenetic factors determine inter-individual variations in the response to diet/nutrition. The latter include inter-individuals’ differences in metabolic functions, including those regulating the absorption, bioavailability, metabolism of nutrients and the metabolic function of organs/cells [8,9,10,11,12,13,14]. Recent data suggest that skeletal muscle mass and specifically muscle fibers exhibit an important role, through their fiber-type depending metabolic functions [15,16,17,18,19,20,21] or their contraction-mediated secretion of myokines [22,23,24,25,26]. For example, it has been recently reported that the total energy expenditure per lean body mass (LBM)—and not nutrition itself—is adolescents’ primary determinant of body composition, as both normal weight and obese adolescents tend to receive increased total energy intake, while at the same time exhibit poor nutritional habits [27]. Accordingly, it has been recently proposed that lipidemic blood profile is mainly determined by how humans and animals metabolize their dietary intake, and not by their nutrition per se, while it has been hypothesized that individuals possessing greater proportions and sizes of oxidative muscle fibers should also be characterized by healthier lipidemic profiles, compared to individuals with increased glycolytic fibers, even if both follow similar nutritional plans [14].

Regarding bioenergetics, glucose and lipids metabolism is upregulated during endurance exercise [28,29,30,31,32,33]. Additionally, long-term, regular endurance training results in volume (total distance or training duration per week) dependent, beneficial and specific adaptations on glucose and lipids metabolism, leading to completely different metabolic procedures and functionalities of glucose and lipids metabolic pathways than those observed in sedentary individuals [28,34,35,36,37,38]. For example, according to the “athletes’ paradox” phenomenon, even though endurance athletes possess increased intramuscular lipids concentrations, their insulin sensitivity is enhanced, a phenomenon attributed to the high daily metabolic demands during and after training bouts but primarily to the training-specific adaptions in oxidative capacities of their muscle fibers [15]. In contrast, nutritionally derived downregulation of oxidative, glucose and lipids metabolism seems to be the main cause of insulin resistance, obesity, dyslipidemia, and metabolic abnormalities development in sedentary individuals [14]. In animals [16,17,18] and humans [19,20,21] with increased muscle fibers’ oxidative capacities, long-term high-caloric or high-fat nutrition (total calories derived from >30% fats, 45–60% carbohydrates, 10–20% proteins; [7,39]), seems to result in lower adverse effects on their body/fat mass and insulin resistance. The latter suggests a “protective mechanism” of muscle fibers’ increased oxidative capacities against the negative impact of high caloric/fat nutrition on body composition and insulin resistance [16,17,18,19,20,21].

Based on the above, it could be suggested that as endurance athletes are characterized by higher proportions and sizes of oxidative muscle fibers [40,41], while their regular, high-volume endurance training leads to greater energy expenditure [42,43,44,45] and to volume dose-depended mitochondrial/oxidative capacities adaptations [37,46], the negative impact of unhealthy high-fat nutrition on their body composition, blood glucose and lipidemic indices concentrations will be of a lower extent, while their nutritional habits will not be the determinant parameters of their body composition, blood glucose concentration and lipidemic profile. On the contrary, the major determinant parameters of sedentary individuals’ body composition, blood glucose concentration and lipidemic profile should be their nutritional habits. The latter is supported by the fact that sedentary individuals do not exhibit the “protective mechanism” derived from the high-volume endurance training-specific metabolic adaptations mentioned above [2,4,5,6]. However, at least to our knowledge, this hypothesis remains unexamined, particularly in middle-aged males, who are more likely to develop chronic cardiovascular diseases compared to young and elderly males [47].

Thus, the present study aimed to investigate the main determinant parameters of body composition, blood glucose and lipids concentrations between middle-aged sedentary and well-trained endurance individuals, following an unhealthy high-fat diet.

## 2. Materials and Methods

### 2.1. Experimental Approach to the Problem

The selection of sedentary and well-trained individuals was based on the purpose of the present study, aiming to compare which parameters could be the major determinants of body composition, blood glucose and lipids concentrations between two extremely different populations: middle-aged sedentary individuals (who do not exhibit the previously mentioned metabolic adaptations) and well-trained endurance athletes (who exhibit these adaptations). Furthermore, in order to investigate if the endurance-specific training-induced metabolic adaptations may act as a “protective mechanism” against the negative impact of unhealthy nutrition on body composition, blood glucose and lipids concentrations, only participants who follow unhealthy high-fat (but not low-very low carbohydrate/ketogenic) diets for a long time were included in the present study, as this type of diets exhibits the most pronounced adverse effects on body composition, blood glucose and lipids concentrations [2,4,5,6]. Thus, according to the above, participants of the two groups should not necessarily match regarding their body composition, energy expenditure etc. However, their age and nutritional intake should be similar. In this cross-sectional study, participants were recruited from the local hospital facilities and athletic clubs. The inclusion criteria were: (1) only male participants, (2) age range between 45 to 65 years, (3) participants of the sedentary group should not fulfil the ACSM’s recommendation for active physical activity engagement [48], while participants of the Runners group should be trained regularly and participate in marathon and ultra-marathon races for at least ten years and be in their preparation period, (4) absence of restraining orthopedic/neuromuscular disorders, (5) absence of drugs abuse or medication administration which are known to affect the neuromuscular system, glycemic and lipidemic blood profiles and body composition (participants of the sedentary group, were recruited from the local hospital, during their annual checkup visit, and before the initiation of any medication treatment, if the latter was medically indicated), (6) no supplement consumption, (7) all participants should follow an unhealthy high-fat diet (30–40% fats, >10% of daily energy intake from saturated fatty acids, 45–60% carbohydrates, 10–20% proteins) [7,39] but not very low carbohydrate/ketogenic diets (<total calories derived from >45% fats, <40% carbohydrates, 10–30% proteins) [49] and (8) abstain from weight loss or maintenance dietary plans. The inclusion of middle-aged marathon and ultramarathon runners was based on recent studies reporting that despite their regular training engagement, they do not seem to follow healthy nutritional habits as they consume high amounts of fats [43,45]. Responders visited the laboratory two times. During the first visit, medical screening by a trained physician and body composition evaluation through bioelectrical impedance analysis (BIA) were performed. Additionally, a trained-registered dietician instructed all participants (detailed oral and written consulting) on how to record their nutritional intake habits for the following seven days. The formal 7-day nutritional recall questionnaires used in the present study were completed at the end of the seven days (visit 2) by each participant in the presence of our trained-registered dietician [42,43,50]. During the 7 days mentioned above, daily energy expenditure was evaluated by activity trackers provided to each participant during the first visit and returned on the second visit [42]. Finally, during the second visit, fasting blood samples were obtained. This study was conducted according to the guidelines of the Declaration of Helsinki, and the Research Ethics Committee of the Department of Nutrition Sciences and Dietetics, Faculty of Health Sciences, International Hellenic University (ID Code: 2.1/989/20.01/2021, date of approval: 9 February 2021), approved all procedures involving human subjects/patients. At the same time, all participants signed informed consent before entering the research procedure.

### 2.2. Participants

Of the initially 120 responders, 71 fulfilled the inclusion criteria. Volunteers were allocated into two groups: (A) Sedentary (Sed; *n* = 35; Age: 54.0 ± 6.6 years, Body Mass: 85.8 ± 13.5 kg, Body Height: 1.8 ± 0.1 m, BMI: 31.3 ± 6.0 kg·m^−2^, Weekly Physical Activity: 75.5 ± 10.9 min of low to moderate-intensity aerobic activity) and (B) Runners (Run; *n* = 36; Age: 51.6 ± 5.2 yrs, Body Mass: 77.1 ± 10.5 kg, Body Height: 1.7 ± 0.1 m, BMI: 23.2 ± 1.8 kg·m^−2^, Weekly training Sessions: 5.9 ± 1.0, Training session Duration: 78.4 ± 28.2 min, Weekly Endurance Training Workload: 89.5 ± 28.6 km of moderate to high intensity, Endurance Training Background: 18.6 ± 12.9 years).

### 2.3. Procedures

#### 2.3.1. Anthropometric Characteristics and Body Composition

Height was measured using a stadiometer (SECA 220, Seca Corporation, Chino, CA, USA), while body mass using a calibrated digital scale (Seca 707, Seca Corporation, Columbia, USA). Body composition was evaluated via BIA (50 Hz; Bodystat 1500, Bodystat Ltd. Cronkbourne, Douglas, Isle of Man), and measurements were analyzed using Bodystat 1500 computer software, with the use of corresponding equations for Caucasian male and female participants (Bodystat 1500 Body Manager, version3.16, 2002, Bodystat Ltd.), according to manufacturer instructions [The intraclass correlation coefficient (ICC) ranged between 0.93 and 0.98].

#### 2.3.2. Evaluation of Dietary Intake, Physical Activity and Energy Expenditures

Food models were employed to estimate the consumed food quantities. Furthermore, an experienced dietitian analyzed food diaries via the Food Processor Program (version 7.4, ESHA Research, Salem, OR, USA), including local-traditional food references [42,43,44,50]. ICC ranged from 0.850 to 0.901. Participants’ physical activity and energy expenditure during the 7-day recording period were evaluated through Garmin Vivoactive 3 smartwatch activity tracker (Digital Tracer Electronics S.A., Alimos, Attika, Greece; ICC: 0.92) [42,43]. Resting metabolic rate (RMR) and respiratory exchange ratio (RER), were evaluated through indirect calorimetry (Vmax 229D, Sensormedics, Yorba Linda, CA, USA), with participants in a prone position for 20 min [50] (ICC: 0.897; 95% CI: Lower = 0.85, Upper = 0.94; *p* < 0.0001, *n* = 10).

#### 2.3.3. Blood Sampling and Biochemical Assays

Venous blood samples were obtained from each participant after overnight fasting. Complete blood count was determined in EDTA anticoagulated whole blood sample on a Mindray BC-3000 haematology analyzer (Mindray, Shenzhen, China). For biochemical parameters’ determination, blood samples were drawn in Vacutainer-type tubes containing a clot activator and centrifuged for 30 min. The recovered sera were stored at −40 °C until further analysis. Serum glucose (Glu), triglycerides (TG), total cholesterol (TC) and High-density lipoprotein (HDL-C) concentrations were measured through a Mindray BS-300 Chemistry Analyzer (Mindray, Shenzhen, China). Low-density lipoprotein (LDL-C) was calculated according to the Friedewald equation. LDL-C/HDL-c and Total Cholesterol/HDL-C ratios were also calculated.

### 2.4. Statistical Analyses

A Shapiro-Wilks test was used to assess the normality of our data. No normality violations in distribution were found (*p* > 0.05). All data are presented as means ± SD. Independent sample *t*-test was employed for the identification of group differences. Cohen’s d, in absolute values, was also calculated as an effect size indicator. Pearson’s product-moment correlation coefficient was used to explore correlations between variables. The interpretation of the observed correlations was performed according to Hopkins’ ranking: correlation coefficients between 0.3–0.5 were considered moderate, between 0.51–0.70 large, between 0.71–0.90 very large, and >0.91 almost perfect. Multiple regression analyses (stepwise) were used to determine the best linear combination and selective predictors’ relative strengths on the determination/explanation of body composition, glycemic and lipidemic blood indices. Statistical analyses were performed with SPSS Statistics Ver. 20 (IBM Corporation, Armonk, ΝY, USA). *p* ≤ 0.05 was used as a 2-tailed level of significance.

## 3. Results

As expected, significant differences in all body composition parameters were found between the groups, with the Run group exhibiting lower body and fat mass and higher LBM (*p* < 0.001; Table 1). No significant differences between the groups were found regarding their nutritional intake (*p* > 0.05; Table 1). The Run group presented significantly higher daily energy expenditure RMR, lower RER, and higher negative energy balance compared to the Sed group (*p* < 0.001; Table 1), observations which are commonly reported in endurance athletes during the preparation period [42,43,44,45,51,52]. As expected, significant differences between the groups were also found for all blood indices, with individuals of the Run group exhibiting healthier glucose and lipids concentrations compared to the Sed group (*p* < 0.001; Table 1).

When all participants were included as one group, moderate correlations were found between daily energy intake, expenditure, energy balance and body and fat masses, Glu, TC, TG, HDL-C and LDL-C concentrations (r: −0.589–0.679, *p* < 0.05; Table 2). However, when these correlation analyses were performed separately for each group, different correlations were found for the correlated variables and their magnitude (Table 2). In the Run group, the only significant correlations (moderate to large) were those between body, fat mass, Glu, TC, TG, HDL-C and LDL-C concentrations and weekly endurance workload, energy expenditure and energy balance (r: −0.796–0.769, *p* < 0.05; Table 2), while no significant correlations were found for total energy and macronutrients intake (*p* > 0.05). In contrast, in the Sed group, the only significant correlations were those for total energy and macronutrient intake (r: 0.418–0.794, *p* < 0.05; Table 2). Finally, total energy expenditure was highly related to the endurance training workload in the Run group (r: 0.803, *p*: 0.001).

The present study’s most important and novel finding was the results of multiple linear regression analyses. According to the assumptions of the linear regression analysis, total energy intake, expenditure, fat and carbohydrate intake (kcal) were included in the analyses. The results are presented in detail in Table 3 (Variance Inflation Factor values ranged from 1.165–3.000; Tolerance values ranged from 0.276 to 0.999). Briefly, body composition, blood glucose and lipids concentrations in the Run group were primarily determined by the energy expenditure (B coefficients ranged between −0.879 and −1.254) and, to a lesser extent, by total energy intake (B coefficients ranged between −0.499 and 0.511), compared to the Sed group, in which the above parameters were primarily determined by total fats and carbohydrates energy intake (B coefficients ranged between −0.754 and 0.724).

## 4. Discussion

The main finding of the present study was that body composition, blood glucose, and lipids concentrations seem to rely on different determinants between middle-aged sedentary and well-trained endurance individuals. In sedentary middle-aged individuals, body composition, blood glucose, and lipids concentrations seem to be determined by their nutritional intake per se. In contrast, body composition, blood glucose and lipid concentrations of well-trained endurance middle-aged individuals are primarily determined by the amount of their energy expenditure (which in turn is determined by the endurance training workload) and, to a lesser extent, by their daily total energy intake, while fat, carbohydrates and proteins intake per se seems not to be a determinant factor in these individuals.

According to the present study’s results, the negative impact and/or the contribution/determination of a high high-fat diet on body composition, blood glucose and lipids concentrations is not the same between middle-aged sedentary and well-trained endurance individuals. Probably, the results presented herein are the outcomes of endurance athletes’ metabolic demands during their long-term, systematic, high-volume endurance training, leading to significantly increased amounts of glucose and lipids, which are utilized to cover their high training-induced metabolic stress [37,38,53,54], but mostly of their training-specific metabolic adaptations in their muscle fibers [14,28,34,35,36,40,46]. High training volumes lead to increased energy expenditure and thus to higher metabolic stress during training [37,38], which increases the percentage area of muscle occupied (%CSA) by Type I and IIa muscle fibers in a dose-dependent manner [40,41,55,56]. Therefore, well-trained endurance athletes exhibit a significantly higher %CSA of Type I muscle fibers [40,41] contrary to the sedentary, obese, diabetic individuals and the ones with significant metabolic dysfunctions which are characterized by low daily energy expenditure and increased proportions of type IIx (glycolytic) muscle fibers [14,20,56,57,58,59,60]. Furthermore, endurance athletes’ regular high-volume training leads to dose-dependent increases in mitochondria content and mitochondria oxidative capacities [29,46,61]. These adaptations, in turn, lead to differential regulations of glucose and lipids metabolism in their oxidative muscle fibers, in terms of the amount of glucose and lipids utilization and functionality of the muscle fibers’ molecular cascades regulating these utilizations, compared to sedentary individuals [14,37,62,63,64,65]. These adaptations seem to make endurance athletes metabolically more flexible [14,66]. According to the concept of “metabolic flexibility”, individuals who can use higher amounts of lipids as their main energy source during the day, therefore exhibiting low RER values during rest, are those with the lowest possibility for the development of obesity, insulin resistance, and dyslipidemia [67,68], independently of their daily total energy intake and diet’s macronutrients composition [68]. However, low RER values are expected to be found in individuals with high proportions and sizes of Type I muscle fibers, and not in those with high proportions and sizes of glycolytic muscle fibers [59]. Indeed, the well-trained runners of the present study, which are expected to have increased proportions and sizes of Type I muscle fibers [40,41], exhibited significantly lower resting RER values, indicating greater lipids utilization during rest to cover their higher resting/total metabolic needs. On the contrary, sedentary obese individuals are expected to have increased proportions and sizes of glycolytic muscle fibers [20,56,57,58,59,60]. Furthermore, the above-mentioned training-induced adaptations in muscle fiber composition and their metabolic properties in well-trained endurance athletes should act as a “protective mechanism” against the high caloric/fat diet-induced obesity, insulin resistance and dyslipidemia [14,16,17,18,19,20,21]. Lean individuals, mostly those with high proportions, sizes and metabolic properties of Type I muscle fibers, exhibit stronger up-regulation of their oxidative muscle fibers’ molecular cascades [62,69] and genes linked with lipids, glucose utilization/oxidation and mitochondria functions [70]. Consequently, they can utilize the nutrient-derived high amount of carbohydrates and lipids more efficiently, minimizing these diets’ negative effects on body composition and metabolism, which is further evidence of metabolic flexibility [14,70]. Indeed, animals and humans with increased proportions, sizes and metabolic properties of oxidative muscle fibers seem to be protected from the long-term poor and/or high-fat nutrition/diets induced obesity and metabolic dysfunctions, while those with low Type I muscle fibers (but mostly those with poor metabolic properties of their muscle fibers) seem to be affected in a greater extent by the poor nutritional habits [16,17,71,72]. In support, it has been recently reported that the high-volume training-induced adaptations in muscle fiber composition, body composition, glycemic and lipidemic blood profiles were highly related to individuals experiencing the highest increases in their Type I %CSA, and thus of their metabolic properties. These individuals had the most beneficial changes in their body composition and glycemic and lipidemic blood profiles [56]. Furthermore, the above-mentioned high-volume training-induced adaptations were not affected/determined by the nutritional habits of these individuals [56]. Considering all the above altogether, it seems that the training-induced adaptations in individuals with long-term high-volume systematic endurance training make them more resistant to the negative impact of a high-fat diet on their body composition, blood glucose and lipids concentrations and minimize the importance/contribution of the high-fat diets on the above cardiovascular risk factors. The results of the present study provide further support to the recently proposed important role of skeletal muscle and mostly of muscle fibers on blood lipidemic profile regulation [14]

However, to avoid any misinformation or misunderstanding, it must be pointed out that the present results do not indicate that the adverse effects of long-termed high-fat nutrition are eliminated in individuals performing regularly endurance training. Even if the participants of the Run group systematically performed (for almost 20 years) high-volume endurance training, exhibited increased daily energy expenditure and a significant negative energy balance due to their unhealthy nutritional habits, their glycemic and lipidemic blood concentrations were within the normal range but at the upper limits. It has been reported that even in inactive animals with increased proportions, sizes and oxidative capacities of Type I muscle fibers, which seem to be a “protective mechanism” against the high caloric/fat diet-induced obesity, insulin resistance and dyslipidemia [16,17,18,19], in very long term high caloric/fat diets, the proportion but mostly the metabolic properties of their Type I muscle fibers decreased significantly. As a result the “protective mechanism” seems to disappear slowly, concomitant with the increase in glycolytic muscle fibers [73]. This may be the reason for the non-optimal glucose and lipids blood concentration levels observed in the well-trained middle-aged runners of the present study, even if endurance training is known to favor healthy body composition and glucose-lipids blood concentrations [74]. However, according to the results of the present study, it seems that the regular high-volume training of our endurance middle-aged athletes lowers the negative effect of a long-term high-fat diet. Thus, the present study does not negate the well-known adverse effects of poor/unhealthy nutrition on cardiovascular risk factors. Nor it suggests that individuals following high-fat diets would eliminate the malnutrition’s adverse effects when enrolling in systematic high-volume endurance training. It just indicates that body composition, blood glucose, and lipid concentrations of well-trained endurance individuals have different determinants compared to middle-aged sedentary individuals, probably due to the high volume of training-induced specific metabolic stress and adaptations.

Even if the present study has several strong points, including the strict inclusion criteria and the methodological procedures that were followed etc., it also has some limitations. The first limitation is the number of participants in each group and the lack of other age, sex and lifestyle choices groups (young/old individuals, female participants, moderate/highly active individuals, individuals with different nutritional habits, patients etc.). Secondly, it was a cross-sectional, observational study and not an interventional study in terms of physical training and/or nutrition, in which the training and nutritional responses and adaptations could be monitored throughout the study time course. Finally, the most important limitation of the present cross-sectional study was that the biochemical-molecular background that could explain our results could not be investigated. However, based on the existing literature, a strong physiological explanation has been provided. Future studies should address the above limitations of the present study.

## 5. Conclusions

In conclusion, the results of the present study suggest that in well-trained endurance middle-aged athletes, body composition, blood glucose and lipids concentrations are determined by their training-induced daily energy expenditure, which alongside the beneficial training-induced metabolic adaptations also leads to an energy deficit and not by their nutritional intake per se, while nutrition is the primary determinant in aged-matched sedentary individuals, even if they both follow high-fat diets. The results of the present study provide further support to the new dogma “Sitting is the new smoking” [75], indicating that a sedentary lifestyle is the number one risk factor nowadays for the development of several cardiometabolic health-related chronic issues, and not the unhealthy nutritional habits per se. Therefore, in clinical practice, health professionals should encourage their patients not only to increase their physical activity but to participate in systematic, long-term exercise programs specifically designed to induce increased energy expenditure, which in turn will lead to the necessary adaptations in skeletal muscles’ and muscle fibers’ metabolic properties, to counterbalance the negative impact of the modern nutritional habits.

## Figures and Tables

**Table 1:** Participants characteristics (All data are presented as means ± SD).

	Runners (*n* = 36)	Sedentary (*n* = 35)	Cohen’s d/*p*
**Body Composition**			
**Body Mass (kg)**	77.10 ± 10.54	85.84 ± 13.46	0.832/<0.001
**Body Mass Index (kg·m^−2^)**	23.20 ± 1.79	31.30 ± 6.02	1.353/<0.001
**Body Fat Percentage (%)**	16.71 ± 3.20	36.34 ± 4.02	5.400/<0.001
**Body Fat Mass (Kg)**	12.88 ± 5.81	31.67 ± 10.22	2.277/<0.001
**Body Fat Mass Index (kg·m^−2^)**	4.23 ± 1.91	11.56 ± 3.41	2.667/<0.001
**Lean Body Mass**	64.21 ± 7.98	54.16 ± 10.01	1.113/<0.001
**Lean Body Mass Index (kg·m^−2^)**	22.08 ± 1.79	18.74 ± 2.78	0.573/0.009
**Energy Balance and Nutritional Evaluation of Macronutrient Components**			
**Energy Intake (kcal)**	2685.12 ± 397.44	2649.59 ± 521.20	0.076/0.910
**Resting Metabolic Rate (kcal)**	1889.82 ± 295.20	1356.1 ± 226.62	2.024/0.010
**Energy Expenditure (kcal)**	3001.88 ± 445.19	2391.25 ± 522.89	1.258/<0.001
**Energy Balance (kcal)**	−316.75 ± 178.18	258.33 ± 175.79	3.248/<0.001
**Fats as Percentage of Total Calories Intake (%)**	35.09 ± 3.12	34.00 ± 3.91	0.065/0.333
**Fats Energy Intake (kcal)**	939.72 ± 261.81	900.86 ± 249.84	0.145/0.414
**Percentage of Saturated Fatty Acids of Total Energy Intake (%)**	14.07 ± 2.8	14.85 ± 2.1	0.075/0.525
**Percentage of Monounsaturated Fatty Acids of Total Energy Intake (%)**	16.08 ± 4.2	15.28 ± 4.9	0.047/0.725
**Percentage of Polyunsaturated Fatty Acids of Total Energy Intake (%)**	4.69 ± 3.12	4.72 ± 3.02	0.035/0.740
**Carbohydrates as Percentage of Total Calories Intake (%)**	51.70 ± 6.87	54.22 ± 5.79	0.096/0.505
**Carbohydrates Energy Intake (kcal)**	1396.26 ± 248.62	1430.77 ± 355.72	0.112/0.103
**Proteins as Percentage of Total Calories Intake (%)**	13.28 ± 4.07	12.11 ± 4.24	0.081/0.131
**Proteins Energy Intake (kcal)**	349.06 ± 120.94	317.91± 93.25	0.287/0.067
**Resting Respiratory Exchange Ratio**	0.80 ± 0.08	0.88 ± 0.06	1.129/<0.001
**Blood Glucose and Lipidemic Profiles**			
**Glucose (mg·dL^−1^)**	85.33 ± 10.91	110.85 ± 13.25	2.108/<0.001
**Total Cholesterol (mg∙dL^−1^)**	186.36 ± 35.37	251.20 ± 40.58	1.706/<0.001
**Triglycerides (mg∙dL^−1^)**	128.21 ± 29.17	229.75 ± 60.98	2.144/<0.001
**High-Density Lipoprotein (mg∙dL^−1^)**	62.48 ± 8.46	36.70 ± 7.82	3.160/<0.001
**Low-Density Lipoprotein (mg∙dL^−1^)**	98.28 ± 25.18	168.57 ± 55.97	1.635/<0.001
**Low to High-Density Lipoprotein Ratio**	1.77 ± 1.14	4.58 ± 2.75	1.341/<0.001
**Total Cholesterol to High-Density Lipoprotein Ratio**	2.98 ± 1.44	6.83 ± 2.93	1.675/<0.001

**Table 2:** Correlations (Pearson’s r) between body, fat masses, blood glucose, total cholesterol, triglycerides, High-density lipoprotein (HDL-C), Low-density lipoprotein and weekly endurance training workload, energy balance and macronutrients intake. Only the significant correlations are presented.

	Body Composition		Resting Blood Indices				
	Body Mass	Body Fat Mass	Glucose	Total Cholesterol	Triglycerides	High-Density Lipoprotein	Low-Density Lipoprotein
**All Participants (*n* = 71)**							
**Weekly Endurance Workload**							
**Energy Intake**	0.510	0.485 *	0.325 *	0.489 *	0.398 *		0.470 *
**Energy Expenditure**	−0.570	−0.548	−0.425 *	−0.396 *	−0.448 *		−0.589
**Energy Balance**	0.487 *	0.522		0.679	0.572	−0.468 *	0.609
**Fats Energy Intake**							
**Carbohydrates Energy Intake**							
**Proteins Energy Intake**							
**Runners (*n* = 36)**							
**Weekly Endurance Workload**	−0.702	−0.752	−0.666	−0.746	−0.778	0.761	−0.796
**Energy Intake**							
**Energy Expenditure**	−0.600	−0.688	−0.600	−0.552 *	−0.598 *	0.584 *	−0.622
**Energy Balance**	0.666	0.570	0.658	0.717	0.769	−0.721	0.758
**Fats Energy Intake**							
**Carbohydrates Energy Intake**							
**Proteins Energy Intake**							
**Sedentary (*n* = 35)**							
**Weekly Endurance Workload**							
**Energy Intake**	0.702	0.794	0.455*	0.657	0.574 *		0.666
**Energy Expenditure**							
**Energy Balance**							
**Fats Energy Intake**	0.500 *	0.589 *		0.494 *	0.503 *		0.469 *
**Carbohydrates Energy Intake**	0.387 *	0.505 *	0.449 *	0.418 *	0.511 *		0.538 *
**Proteins Energy Intake**							

*p* < 0.001 except when r values are marked with (*) in which *p* < 0.05.

**Table 3:** Beta coefficients (B) as indicators of the selected predictors’ relative strengths/impact on body, fat masses, blood glucose, total cholesterol, triglycerides, High-density lipoprotein (HDL-C), Low-density lipoprotein, in each group separately.

		Body Composition		Resting Blood Indices				
		Body Mass	Body Fat Mass	Glucose	Total Cholesterol	Triglycerides	High-Density Lipoprotein	Low-Density Lipoprotein
**Runners (*n* = 36)**								
**R/*p***		0.600/<0.001	0.724/<0.001	0.654/<0.001	0.720/<0.001	0.769 <0.001	0.772/<0.001	0.749/<0.001
**Energy Intake**	B	0.400	0.511	0.358	0.465	0.497	−0.499	0.500
	*p*	0.020	0.013	0.042	0.010	0.017	0.017	0.014
**Energy Expenditure**	B	−0.982	−0.962	−0.879	−1.166	−1.237	1.229	−1.254
	*p*	<0.001	<0.001	<0.001	<0.001	<0.001	<0.001	<0.001
**Fats Energy Intake**	B							
	*p*							
**Carbohydrates Energy Intake**	B							
	*p*							
**Sedentary (*n* = 35)**								
**R/*p***		0.505/0.002	0.666/<0.001	0.589/<0.001	0.657/<0.001	0.613/<0.001	0.706/<0.001	0.666/<0.001
**Energy Intake**	B	0.682	0.437	0.724	0.600	0.722	−0.754	0.641
	*p*	0.001	0.010	<0.001	<0.001	<0.001	<0.001	<0.001
**Energy Expenditure**	B							
	*p*							
**Fats Energy Intake**	B	0.328	0.502	0.478	0.610	0.589	−0.625	0.608
	*p*	0.022	0.020	0.008	<0.001	0.009	<0.001	<0.001
**Carbohydrates Energy Intake**	B	0.444	0.432	0.689	0.500	0.342	0.509	0.444
	*p*	0.009	0.011	<0.001	0.020	0.048	<0.001	0.025

## Data Availability

Available after a reasonable request from the corresponding author.

## Abbreviations

%CSA: Percentage area of muscle occupied by each muscle fiber type; ACSM: American College of Sports Med.; B: Beta coefficient of multiple regression analyses; BMI: Body mass index; EDTA: Ethylenediamine tetraacetic acid; Glu: Serum glucose; HDL-C: Serum high-density lipoprotein; ICC: intraclass correlation coefficient; LBM: Lean body mass; LDL-C: Serum low-density lipoprotein; RER: Respiratory exchange ratio; RMR: Resting metabolic rate; Run: Runners group; Sed: Sedentary individuals group; TC: Total serum cholesterol; TG: Serum triglycerides; Type I: Slow, oxidative muscle fibers; Type IIa: Fast, glycolytic -oxidative muscle fibers; Type IIx: Very fast, glycolytic muscle fibers.
